# Upconversion photoluminescence of Ho^3+^-Yb^3+^ doped barium titanate nanocrystallites: Optical tools for structural phase detection and temperature probing

**DOI:** 10.1038/s41598-020-65149-z

**Published:** 2020-05-29

**Authors:** Manoj Kumar Mahata, Tristan Koppe, Kaushal Kumar, Hans Hofsäss, Ulrich Vetter

**Affiliations:** 1 Second Institute of Physics, University of Göttingen, Friedrich-Hund-Platz 1, 37077 Göttingen, Germany; 2 Department of Physics, Indian Institute of Technology (Indian School of Mines), Dhanbad, 826004 India

**Keywords:** Optical physics, Photonic crystals, Chemical physics, Energy transfer, Optical spectroscopy, Nonlinear optics

## Abstract

Authors have explored the photo-physical properties of Ho^3+^-Yb^3+^ doped BaTiO_3_ nanocrystals and proposed an intuitive method to probe temperature and crystal phase structure of the matrix. Structural phase change of doped crystals was analyzed in terms of their X-ray diffraction, and it was confirmed through second harmonic generation. We give insights on upconversion of energy of light-emission in Ho^3+^-Yb^3+^: BaTiO_3_ nanocrystals upon a 980 nm laser-light excitation and subsequently, the excited state dynamics were studied with the help of dependence of upconversion luminescence on excitation power and measuring-temperature. To understand the nature of occupancies of the Ho^3+^ ions at the Ti- and Ba-sites, we performed site-selective, time-resolved spectroscopic measurements at various crystal phases. Based on the lifetime analysis, it is inferred that the Ho^3+^ ions are present at two types of sites in barium titanate lattice. One of those is the 6-coordinated Ti-site of low symmetry, while the other one is the 12-coordinated Ba-site of higher symmetry. The upconversion emission of the nanocrystals are found to be temperature-sensitive (12 to 300 K), indicating possible use as a self-referenced temperature probe. An analysis of the temperature dependent emissions from ^5^F_4_ and ^5^S_2_ levels of Ho^3+^ ions, gives a maximum value of temperature sensitivity ~ 0.0095 K^−1^ at 12 K. Furthermore, we observe a sharp change in the luminescence intensity at ~180 K due to a ferroelectric phase change of the sample. The correlation of upconversion luminescence with the results of X-ray diffraction and second harmonic generation at different crystal phases implies that the frequency upconversion may be used as a probe of structural change of the lattice.

## Introduction

Ferroelectric titanates of a perovskite ABO_3_ structure (where A and B are usually divalent and tetravalent cations, respectively) are widely used in many applications due to their excellent dielectric, magnetic and electro-optic properties^1–4^. In general, a perfect perovskite has a cubic unit cell of a simple crystal structure of a CaTiO_3_- mineral perovskite. Here, the tolerance factor (T_f_) governs the nature of distortions from its ideal shape, e.g., a rhombohedral or an orthorhombic GdFeO_3_-type crystal structure is observed on effectively small T_f_ ≤ 1, while polytypic structures turn up at T_f_ > 1. The wide range of physical properties of ABO_3_-type oxides are mainly dependent on the relative sizes of the ions, electronic configuration of the ions, dopants and substituents^5,6^. For example, Pandey *et al*. have investigated structure and electronic/magnetic properties of such oxides BiFeO_3_ − PbTiO_3_, BiFeO_3_-BaTiO_3_, etc. in this series^7,8^. Small ABO_3_ crystallites are particularly used in liquefied petroleum gas sensing and humidity sensing^9–12^.

Further, barium titanate (BaTiO_3_) is an important nonlinear optical material which has numerous applications in light modulation, optical memory storage, optical switching and electro-optic phase modulator^13–16^. Additionally, the high photorefractive sensitivity of barium titanate is beneficial for photonic applications^15,16^. Relation between phase transition and crystallite size of BaTiO_3_ is studied in many theoretical and experimental works^17–20^, which show that five phases, namely, rhombohedral, orthorhombic, tetragonal, cubic and hexagonal appear at different temperatures. A low temperature phase change is reported at ~183 K^21^. Considering radius of a rare-earth ion R^3+^ is within Ba^2+^(1.35 Å) and Ti^4+^(0.68 Å), the Ba^2+^ -site (A-site) is preferred to be occupied by a larger R^3+^ such as Nd^3+^ (1.08 Å) and La^3+^ (1.15 Å), while the intermediate ones such as Er^3+^ (0.89 Å) and Ho^3+^ (0.90 Å) may replace both the A- and B- sites^22,23^. BaTiO_3_ is a good optical host suitable for doping R^3+^ ions due to its low phonon cut-off frequency with excellent chemical and mechanical stability, which suits for upconversion luminescence. On the other hand, upconversion luminescence is an optical non-linear anti-Stokes process, which features conversion of low energy photons into high energy by utilizing the quantum mechanically forbidden 4f → 4f optical transitions of R^3+^ ions while doped in crystallite host materials^24–27^. Over the past decade, researchers have shown extensive range of advanced applications of upconversion nanoparticles, spanning from background noise-free biological imaging, theranostics, drug-delivery to photo-voltaic devices, and photochemical reactions^28–33^. To date, several excellent reviews on R^3+^ doped upconversion luminescence have been published to summarize the progress in this field along with demonstration of advanced applications^34,35^. The long-lived intermediate energy levels of the R^3+^ ions are favorable to achieve unique upconversion emission. The electronic transitions within a 4f^n^ shell of R^3+^ ions greatly rely upon their site-symmetry in the crystal lattice of the host materials due to the difference in sensitivity to the crystal field. Therefore, the R^3+^ ions and their positions in different crystal structures may give distinct photoluminescence properties, and thus the knowledge of crystal structure of a given material may be realized by analyzing its luminescence behavior. Despite this interest, the explanation on the site symmetry of R^3+^ ions in BaTiO_3_ through luminescence measurements and the effect of phase transition on upconversion properties have not been studied in depth. Recently, the phase transition induced by high pressure in Eu^3+^: Bi_2_WO_6_ was investigated by Maczka *et al*. by utilizing the variation of emission intensity in downconversion luminescence measurements of Eu^3+^ ions^36^. Here, Bi_2_WO_6_ undergoes two phase transitions near 3.4 and 6.2 GPa pressure what is it clearly reflects in the Eu^3+^ light-emission. In a separate study, Yao *et al*.^37^ measured luminescence properties in the transition of phase structure of Er^3+^ doped Pb(Mg_1/3_Nb_2/3_O_3_)-PbTiO_3_ from a rhombohedral to a morphotropic phase boundary, consecutively to a tetragonal phase on increasing PbTiO_3_ content in this example. However, none of the earlier studies^36–38^ has taken into consideration studying such materials as a structural probe, and a major concern is that the basic luminescence properties in terms of excited state dynamics at steady-state or time-resolved mode with the crystal phase transition have not been addressed so far.

In this work, we report on the comprehensive upconversion properties of Ho^3+^-Yb^3+^: BaTiO_3_ nanocrystals. We have further characterized the structural phases by using not only the X-ray diffraction (XRD) patterns but also second harmonic generation (SHG). The temperature dependent population variation of ^5^F_4_ and ^5^S_2_ Ho^3+^-levels has been employed for phosphorescence intensity ratio based low-temperature thermometry. The variation of site-occupancy of Ho^3+^ ions in BaTiO_3_ of different phases is also investigated based on time-resolved spectroscopic analysis. This study suggests a general approach for sensibly probing crystal phase of this compound.

## Results and Discussion

### Structural properties

**Figure 1:**
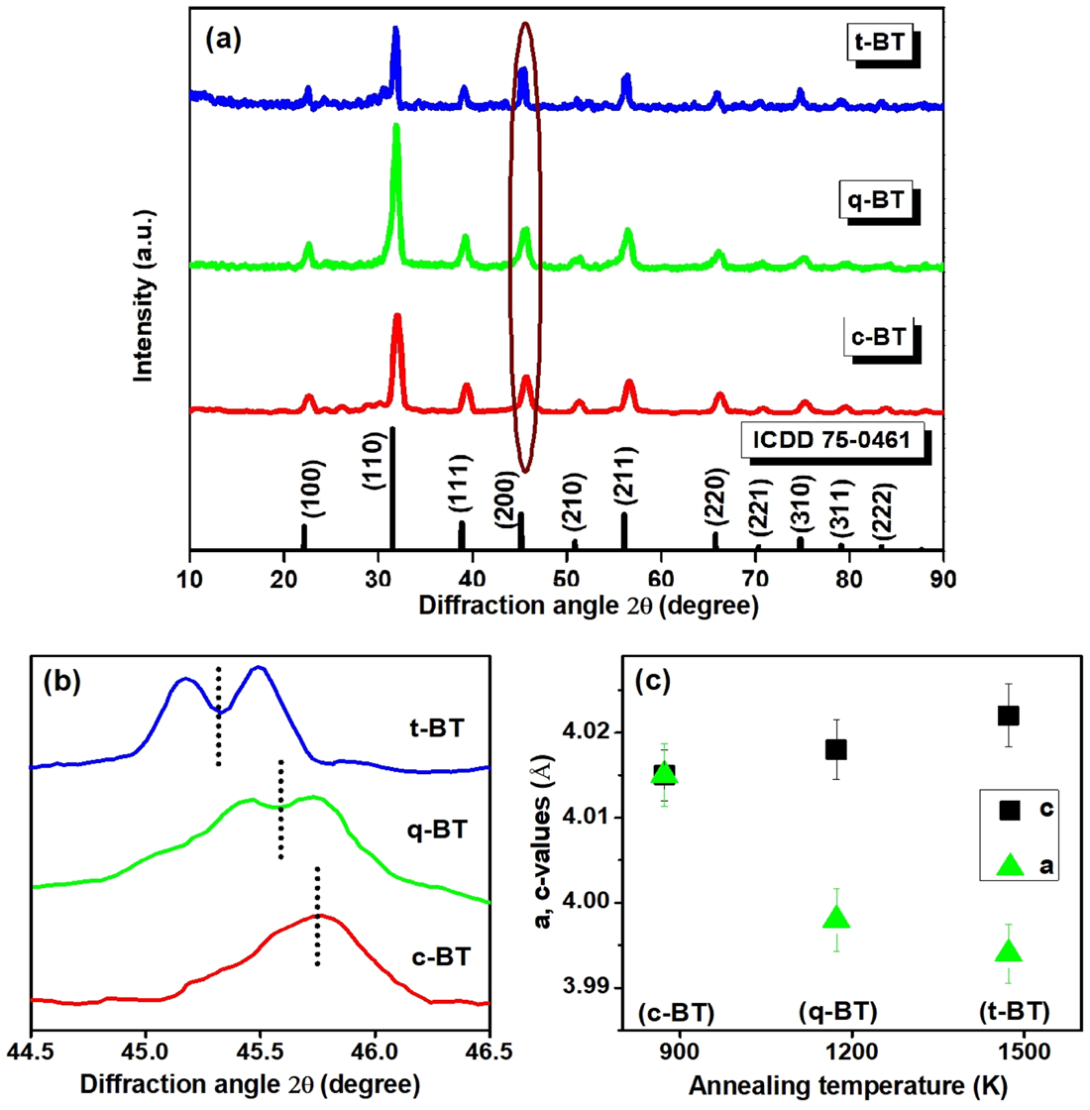
(**a**) XRD patterns of Ho^3+^-Yb^3+^:BaTiO_3_ synthesized at 873 K (c-BT), 1173 K (q-BT) and 1473 K (t-BT), **(b)** magnified XRD peaks around 2θ = 45.71°, and **(c)** variation of lattice parameters with increasing processing temperature, with a c → t-phase transition.

The Ho^3+^-Yb^3^ doped BaTiO_3_ samples, prepared by a wet-chemical precipitation method (*Section*: Methods and analyses), were annealed at three different temperatures 873, 1173 and 1473 K for 6 h and were abbreviated as c-BT (cubic) q-BT (quasi-cubic) and t-BT (tetragonal), respectively, according to their XRD patterns. Figure 1(a) presents the XRD patterns of c-BT, q-BT and t-BT of Ho^3+^-Yb^3+^ doped BaTiO_3_. Following their positions and relative intensities, the individual XRD peaks can be indexed as in the standard cards (ICDD card no. 75-0461) for c- and t-BaTiO_3_ structures^39–41^. The c-BaTiO_3_ structure with the *Pm3m* space group gives lattice parameters *a* = 4.015 Å, while the t-BT sample with space group *P4mm* gives *a* = 3.994 Å and c = 4.022 Å. Tsur *et al*.^22,23^ have reported that Ho^3+^ can occupy both A-site or B-site (known as amphoteric behavior), whereas Yb^3+^ occupies B-site mainly because of its better stability in this site in BaTiO_3_. They have considered thermodynamics and T_f_ value to predict the R^3+^ occupancy in perovskites. An average crystallite size calculated by a well-known Scherrer formula^42^ was found to be 33, 39 and 54 nm (with an error bar ±3 nm) for the c-BT, q-BT and t-BT specimens, respectively. The patterns reveal that an increasing calcination temperature is promoting average crystallite size in a thermally promoted growth of small crystallites. The size of c-phase increased from 33 to 39 nm as the calcination temperature was raised from 873 K to 1173 K. The t-phase exhibits similar result with its crystallite size 54 nm (calcined at 1473 K). However, the crystallite size is unequally bigger in this phase, which is caused due to the fact that it involves a faster diffusion of atoms at so high temperature. That helps an increased formation of nuclei of the t-phase. The grain boundaries, which are among these nuclei, are separated by pores. At so high temperature, the crystallites grow rather rapidly at removal of grain boundaries^43^. A due c → t-BaTiO_3_ transition is demonstrated by splitting of (200) and (202) peaks at 2θ ≈ 45.71°. In the 1473 K sintered sample, two distinct peaks split (Fig. 1b) at 2θ ≈ 45.19° and 2θ ≈ 45.51° in (200) and (002) planes in place of a single (002) peak of c-BaTiO_3_. The specimen prepared at 1173 K owes the onset of t-phase formation. Looking closely to these two peaks, one can readily ascertain the existence of two phases in the q-BT. It is believed that as a result of cell elongation in the c-axis due to a ferroelectric transition at high temperature, the t-phase reforms over the c-phase. Co-existence of crystal phases in a similar perovskite material- barium titanate stannate was reported by Mueller *et al*.^6^ and the phenomenon was interpreted in terms of mechanically clamped c- and t-phases. A phase transition c → t-BaTiO_3_ in a similar range of calcining temperature was reported by Sengodan *et al*.^41^. Consistently, our findings are in this line as put in several reports of synthesis of this material^21,39,40^.

An analysis of XRD patterns on evolution of cubic, quasi-cubic and tetragonal lattice parameters with annealing temperature reveals due shifts in peak positions towards lower 2θ values. This results in a noticeble change of the unit cell volume as sintering temperature is increased from 873 K to 1473 K. What’s more, the effect of annealing temperature on the BaTiO_3_ lattice is reflected in the ratio of their lattice parameters (Fig. 1c). The tetragonality in terms of c/*a* ratio for the three samples- c-BT, q-BT and t-BT are calculated as 1, 1.005 and 1.007, unveiling intriguing correlation of increase in c/*a* ratio with lattice expansion^44^.

The evolution of crystallite size of Ho^3+^-Yb^3+^:BaTiO_3_ with annealing temperature is shown in Figure S1. It increases with increasing annealing temperature. Scanning electron microscopic (SEM) images (Figure S1) reveals that the small cystallites are joined one another in small assemblies. The results are similar to those of Er^3+^-Yb^3+^ doped BaTiO_3_ synthesized using the same procedure in our earlier study^16^. The energy-dispersive X-ray spectra for t-BT and c-BT are shown in Figure S2.

### Optical properties

**Figure 2:**
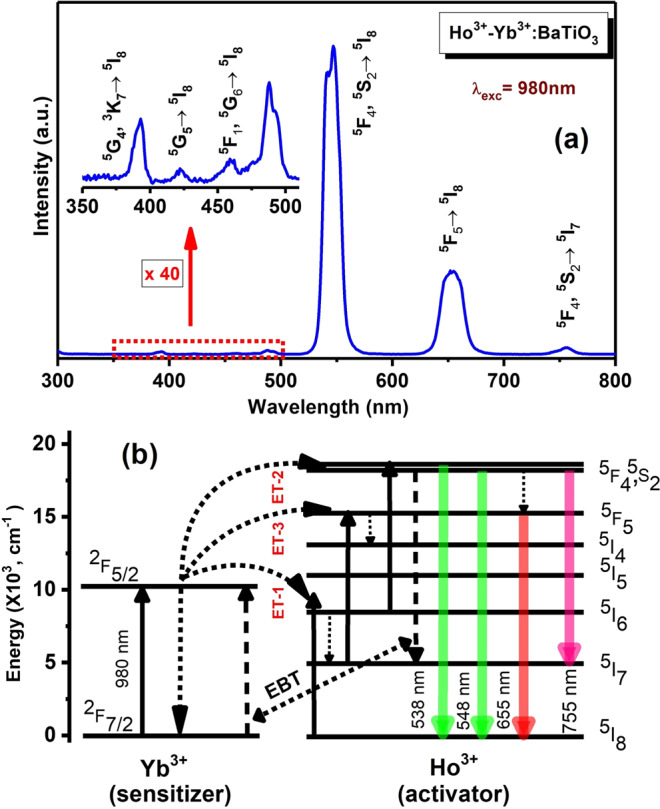
(**a**) Upconversion emission spectrum of Ho^3+^-Yb^3+^: t-BaTiO_3_ recorded at room temperature under 980 nm light excitation and (**b**) an energy-level diagram showing possible transition pathways.

The optical absorption spectrum (Figure S3) of Ho^3+^-Yb^3+^: BaTiO_3_ powder in diffuse reflection mode shows a strong absorption in the Stark components of ^2^F_7/2_ level of Yb^3+^ ions at around 980 nm. The other R^3+^ absorption bands at 524, 655 and 800 nm are attributed to ^5^F_4_, ^5^S_2_ ← ^5^I_8_, ^5^F_5_ ← ^5^I_8_ and ^5^I_5_ ← ^5^I_8_ transitions of Ho^3+^ ions. Upon 980 nm laser-light excitation, the t-BT exhibits intense green (538 and 548 nm) and red (655 nm) frequency upconversions in the ^5^F_4_, ^5^S_2_ → ^5^I_8_ and ^5^F_5_ → ^5^I_8_ transitions, respectively, as shown in Fig. 2a and Figure S4. More details of mechanisms on the upconversion processes and photophysical pathways involved here are provided in the Supplementary Information.

### Laser pulse energy dependent upconversion properties

In order to unravel the nature of upconversion processes, i.e., the number of photons involved in a particular transition in the upconversion emission, the pump power dependent upconversion (Figure S5a) was studied in a simplified model. As known from theoretical analysis, the slope of the logarithmic plot of pump power vs. intensity accounts the number of photons participated in a particular upconversion process^45^. In addition, to understand the mechanism and illustrate the experimental findings in Ho^3+^-Yb^3+^ doped BaTiO_3_, the population mechanism of ^5^F_4_, ^5^S_2_ and ^5^F_5_ levels have been inevitably established by solving the steady-state rate equations at an unsaturated stage of population of the ions. In the present case, the absorption cross-section of Yb^3+^ ions at 980 nm is much higher over the Ho^3+^ value^28^. Therefore, the steady-state rate equations of Ho^3+^-Yb^3+^ in BaTiO_3_, according to Fig. 2b can be set as follows:

1 $$\begin{array}{c}\frac{d{N}_{Ho,1}}{dt}=0={W}_{2}{N}_{Ho,2}+{{W}_{51}}^{\text{'}}{N}_{Ho,5}-{W}_{1}{N}_{Ho,1}-{\rho }_{P}{\sigma }_{14}{N}_{Ho,1}-{K}_{3}{N}_{Ho,1}{N}_{Yb,1}+{W}^{\text{'}}{N}_{Ho,5}{N}_{Yb,0}\end{array}$$

2 $$\begin{array}{c}\frac{d{N}_{Ho,2}}{dt}=0={K}_{1}{N}_{Ho,0}{N}_{Yb,1}+{\rho }_{P}{\sigma }_{02}{N}_{Ho,0}-{W}_{2}{N}_{Ho,2}-{K}_{2}{N}_{Ho,2}{N}_{Yb,1}-{\rho }_{P}{\sigma }_{25}{N}_{Ho,2}\end{array}$$

3 $$\begin{array}{c}\frac{d{N}_{Ho,4}}{dt}=0={W}_{5}{N}_{Ho,5}+{K}_{3}{N}_{Ho,1}{N}_{Yb,1}+{\rho }_{P}{\sigma }_{14}{N}_{Ho,1}-{W}_{4}{N}_{Ho,4}-{W}_{4}^{\text{'}}{N}_{Ho,4}\end{array}$$

4 $$\begin{array}{c}\frac{d{N}_{Ho,5}}{dt}=0={K}_{2}{N}_{Ho,2}{N}_{Yb,1}+{\rho }_{P}{\sigma }_{25}{N}_{Ho,2}-{W}_{5}{N}_{Ho,5}-{W}_{5}^{\text{'}}{N}_{Ho,5}-{{W}_{51}}^{\text{'}}{N}_{Ho,5}\end{array}$$

5 $$\begin{array}{c}\frac{d{N}_{Yb,1}}{dt}=0={\rho }_{P}{\sigma }_{Yb}{N}_{Yb,0}+{W}^{\text{'}}{N}_{Ho,5}{N}_{Yb,0}-({K}_{1}{N}_{Ho,0}+{K}_{2}{N}_{Ho,2}+{K}_{3}{N}_{Ho,1}){N}_{Yb,1}-{W}_{Yb}{N}_{Yb,1}\end{array}$$

where, N_Ho,i_ (i = 0, 1, 2, 3, 4, 5) are the density of population of the levels ^5^I_8_, ^5^I_7_, ^5^I_6_, ^5^I_4_, ^5^F_5_ and ^5^F_4_/^5^S_2_, respectively. The population densities of ^2^F_7/2_ (ground state) and ^2^F_5/2_ (excited state) of Yb^3+^ ions are expressed as N_Yb,i_ (i = 0,1). W_1_, W_2_, W_4_ and W_5_ are the non-radiative decay rates of ^5^I_7_, ^5^I_6_, ^5^F_5_ and ^5^F_4_/^5^S_2_ levels, while the radiative decay rates for the red, green and NIR emissions are expressed as Wʹ_4_, Wʹ_5_ and Wʹ_51_ from the levels ^5^F_5_ and ^5^F_4_/^5^S_2_. The decay rates of Yb^3+^ ions’ ^2^F_5/2_ → ^2^F_7/2_ is W_Yb_, while the energy transfer (ET) rates to Ho^3+^ are K_1_, K_2_ and K_3_ corresponding to ET-1, ET-2, ET-3, respectively, as shown in Fig. 2b. The energy back transfer (EBT) from Ho^3+^ to Yb^3+^ is indicated by using Wʹ as an energy back transfer rate. The absorption cross-section of Ho^3+^ (between ‘i’ and ‘j’) and Yb^3+^ (between ^2^F_5/2_ and ^2^F_7/2_) are represented by σ_ij_ and σ_Yb_, respectively, and the laser pump constant is ρ_P_, which is dependent on incident pumping energy. The Eqs. (1) and (3) are associated with red upconversion emission, while the green upconversion emission is correlated with Eqs. (2) and (4). The frequency upconversion of Ho^3+^-Yb^3+^ is relatively cumbersome due to the involvement of the Ho^3+^ to Yb^3+^ back energy-transfer followed by emission from Yb^3+^. Therefore, the challenges in solving the rate equations are realized by neglecting the less impact terms in the equations. Since the Yb^3+^ ions have much higher absorption cross-section at 980 nm than the Ho^3+^ ions, the terms corresponding to ground state absorption (GSA) and excited state absoprtion (ESA) can be ruled out. In view of the fast multi-phonon relaxation between ^5^I_4_ and ^5^I_5_ levels, the ^5^I_4_ level can be considered as a short-lived level.

When the excitation pulse energy is low, the spontaneous decay of Ho^3+^ ions at the levels ^5^I_6_ and ^5^I_7_ are dominant through the energy-transfer processes ET-2 and ET-3. As a consequence, the relevant terms from Eqs. (2) and (1) can be omitted. Furthermore, the EBT is insignificant compared to the direct photon absorption of 980 nm wavelegth by the Yb^3+^ ions (^2^F_7/2_ to ^2^F_5/2_), allowing us to neglect the relevant term of EBT. From Eq. (5), we get,

6 $${N}_{Yb,1}=\frac{{\rho }_{P}{\sigma }_{Yb}{N}_{Yb,0}}{{K}_{1}{N}_{Ho,0}+{K}_{2}{N}_{Ho,2}+{K}_{3}{N}_{Ho,1}+{W}_{Yb}}.$$

From Eqs. (2) and (4), we consecutively get,

7 $${N}_{Ho,5}=\frac{{K}_{1}{K}_{2}{N}_{Ho,0}}{{W}_{2}({W}_{5}+{W}_{5}^{\text{'}}+{W}_{51}^{\text{'}})}{N}_{Yb,1}^{2}.$$

Using Eqs. (1) and (3), we obtain,

8 $${N}_{Ho,4}=\frac{{W}_{5}{K}_{1}{K}_{2}{N}_{Ho,0}}{{W}_{2}({W}_{4}+{W}_{4}^{\text{'}})({W}_{5}+{W}_{5}^{\text{'}}+{W}_{51}^{\text{'}})}{N}_{Yb,1}^{2}.$$

As the population density (N_Yb,1_) of the level ^2^F_5/2_ of Ho^3+^ ion is proportional to the excitation power density, Eqs. (7) and (8) indicate green and red upconversion emission processes as quadratic power dependent.

On the other way, assuming the dominance of upconversion processes ET-2 and ET-3 over the linear decays at high excitation energy, the relevant terms- W_1_N_Ho,1_ and W_2_N_Ho,2_ can be neglected so to Eqs. (2) and (4) imply,

9 $${N}_{Ho,5}=\frac{{K}_{1}}{({W}_{5}+{W}_{5}^{\text{'}}+{W}_{51}^{\text{'}})}{N}_{Ho,0}{N}_{Yb,1},$$

while Eqs. (1) and (3) consecutively imply,

10 $${N}_{Ho,4}=\frac{{K}_{1}}{({W}_{4}+{W}_{4}^{\text{'}})({W}_{5}+{W}_{5}^{\text{'}}+{W}_{51}^{\text{'}})}[{W}_{5}+{W}_{51}^{\text{'}}]{N}_{Ho,0}{N}_{Yb,1}.$$

Equations (9) and (10) indicate the linear nature of green and red upconversion processes at high pulse energy. However, our experimental observation (Figure S5b) shows the number of participated photons for 548, 655 and 755 nm light emissions as 1.54, 1.61 and 1.60, respectively, which are not directly in complete agreement with the theoretically predicted values. In the present case, a depletion of the ^5^I_6_ and ^5^I_7_ levels occurs due to the upconversion process and linear decay. The experimental observations show the value of involved photon numbers in the upconversion processes within the boundary values 1 and 2. Previous studies have reported that the 548, 655 and 755 nm emission bands are usually due to the absorption of two photons in YVO_4_, Y_2_O_3_, and other luminescent materials^24,28,45,46^.

Further observation on the excitation energy dependent upconversion emission of the bands located at 538 and 548 nm exhibits a systematic behavior of pump power with the ratio of these two bands (I_538_/I_548_) (Figure S5c). This type of variation is usually attributed to laser induced heating of the material, which led us to investigate the material extensively at various measuring-temperatures, as discussed as follows.

### Effect of crystal phase transition on upconversion emission

**Figure 3:**
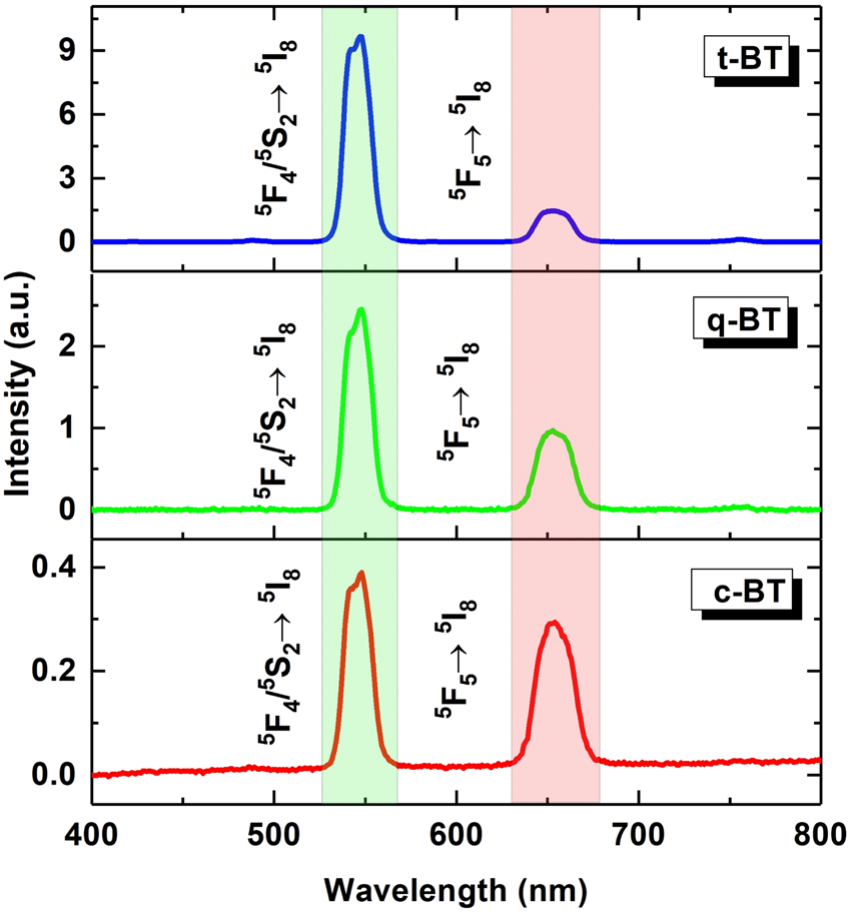
Comparative upconversion spectra upon 980 nm light excitation of Ho^3+^-Yb^3+^: BaTiO_3_ of different phases.

The crystal structure of the doped BaTiO_3_ plays a crucial role in upconversion of energy of its light emission. Electronic transitions of the R^3+^ ions in the 4 f shells are strongly dependent on their site-symmetry in the crystal lattice and the position of the R^3+^ in the lattice is strikingly different in its different crystal structures. Consequently, the information on crystal structure may be derived by deciphering the photoluminescence properties of R^3+^-doped BaTiO_3_ of different structures. In order to realize the possibility of this material as a structural probe, we have thus further examined its 980 nm light  excited frequency upconversion emission properties in its different crystal phases. The comparative upconversion spectra (Fig. 3) show largely enhanced upconversion emission intensity in the c → t-phase transition. Interestingly, the red emission is decreased in intensity over the green one (Figure S6a). In view of the overall emission, the maximum emission intensity is noted for the t-BT phase. Effects of grain-growth and elimination/creation of charge carriers in c-BT, q-BT and t-BT can be responsible for the tailored upconversion emission, wherein the XRD patterns and infrared bands did not indicate countable difference among the samples.

An electronic transition probability is not associated only with the electronic structure but also crystal structure in a solid material. The local R^3+^ crystal fields sensitively vary in its tailored crystal phases, which can promptly tune the electronic transition probability, finely tuning the upconversion emission properties. For example, electronic band-structure dependent frequency upconversion in monoclinic and t-LaVO_4_:Er^3+^ has been studied by Zhang *et al*.^47^, who observed nearly 10-times brighter green emission of t-LaVO_4_:Er^3+^ over its counterpart monoclinic phase, which is evidently due to a difference in the local structure. They have interpreted the origin of tailored light-emission in terms of a tailored local LaVO_4_:Er^3+^ structure. Not many reports are available on the upconversion emission in a phase transition suitable to compare these results more meticulously in terms of tailored charge carriers. Qualitatively, as the structure symmetry decreases in the t-BaTiO_3_, the Ho^3+^ doped sites acquire a larger perturbation field, which in turn arise in an enhanced electronic transition probability in the form of a duly enhanced light emission as observed in this example.

In accordance to the previous section- the solutions of the rate equations at low pump power (LP) and high pump power (HP), the ratios of Eqs. (7) to (8) and Eqs. (9) to (10) can be presented as

11 $${\frac{{I}_{G}}{{I}_{R}}|}_{LP}=\frac{{W}_{5}^{\text{'}}{N}_{Ho,5}}{{W}_{4}^{\text{'}}{N}_{Ho,4}}=\frac{{W}_{5}^{\text{'}}}{{W}_{5}}\left[1+\frac{{W}_{4}}{{W}_{4}^{\text{'}}}\right].$$

and

12 $${\frac{{I}_{G}}{{I}_{R}}|}_{HP}=\frac{{W}_{5}^{\text{'}}}{{W}_{5}+{W}_{51}^{\text{'}}}\left[1+\frac{{W}_{4}}{{W}_{4}^{\text{'}}}\right].$$

In this scenario, a considerable phenomenon is that the Ho^3+^ ions at the ^5^F_4_/^5^S_2_ levels depopulate radiatively at the ^5^I_8_ and ^5^I_7_ levels, producing the green and NIR emission bands and the branching ratio for these two emissions is a significant factor accounting in their intensities. In this context, we have calculated the ratio of green to NIR emission for the three specimens (Figure S6b), which reflects that the intensity increasing in both the emission bands almost linearly and, therefore, branching of Ho^3+^ ions’ populations at the ^5^F_4_/^5^S_2_ level is not responsible for a change in a ratio of green to red emission band (I_G_/I_R_). Thus, based on the results illustrated in the rate equation model, an increased I_G_/I_R_ ratio could be due to several combined effects (i) a decrease of non-radiative decay from ^5^F_4_/^5^S_2_ to ^5^F_5_, (ii) an increase of non-radiative decay from ^5^F_5_ to ^5^I_4_ in a c → t-BaTiO_3_ phase transition, and (iii) an induced crystal field on the Ho^3+^ doped sites.

### Effect of temperature on upconversion emission and temperature sensing

**Figure 4:**
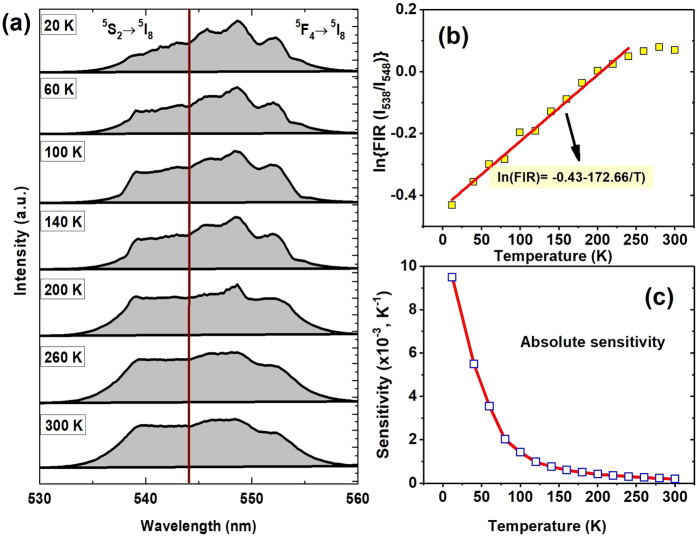
(**a**) Temperature dependent upconversion spectra of t-BT, **(b)** plot of ln(FIR) against temperature, and **(c)** variation of absolute sensitivity with temperature.

In order to understand the Ho^3+^-Yb^3+^:BaTiO_3_ properties at temperatures below room temperature, we have further investigated the upconversion emission as a function of temperature over 12 to 300 K (Fig. 4a). The emission intensity is increased by a large amount at low temperatures due to a deactivation of non-radiative channels. A closer observation of intensities of green and red emissions at selective temperatures reveals that at ~ 180 K, there is a sharp jump in their values, as shown in Figure S7. This abnormal intensity change in these upconversion emission bands at this specific temperature could be associated to a structural change in BaTiO_3_ with its ferroelectric phase transition and Ho^3+^crystal field symmetry is altered in the transition. Recently, Zuo and coworkers^48^ have reported a similar investigation of Er^3+^-emission intensity change at Curie transition temperature (395 K) of the BaTiO_3_ matrix. Figure S7 also illustrates that the upconversion emission from ^5^F_4_ (538 nm) level is notably suppressed, while it is increased remarkably from the ^5^S_2_ (548 nm) level. The ^5^F_4_ state is quenched thermally to ^5^S_2_ what is it contributes an enhanced ^5^S_2_ → ^5^I_8_ emission on cooling the sample. Alternatively, as the temperature increases, the intensities of 538 nm and 548 nm bands tend to be equal at ~ 260 K (Figure S7) and it anticipates that at higher temperature (≥ 300 K), the 538 nm band will be starting raising its intensity on hot phonons, which help to populate Ho^3+^ ions in the ^5^F_4_ level from the ^5^S_2_ level. However, the ratio 538 nm/548 nm (Fig. 4b) is not affected due to the phase transition, which infers that the influence of thermal quenching is more effective than that of the phase transition for the ^5^F_4_/^5^S_2_ levels. Additionally, the temperature dependent emissions from ^5^F_4_ and ^5^S_2_ levels reflect that this material could be utilized for non-contact thermometry. Therefore, to investigate its temperature sensing ability, its temperature dependent upconversion emission spectra were inspected over 12 K to 300 K. The ^5^F_4_ and ^5^S_2_ levels of Ho^3+^ are closely located so as to thermally exchange couple each other. Thus, the variations of relative intensities of these bands were studied at different temperatures. It is observed that the overall intensity increases as the sample cools down with no any shift in the two bands. The ratio of intensities of 538 nm and 548 nm emission bands is seen to be increasing in the sample warming from 12 K to 300 K. The variation of relative intensities of these two emission bands at different temperatures was calculated and the graph is shown in Fig. 4b.

The fluorescence intensity ratio (FIR) of the two bands can be written as^49,50^,

13 $$FIR=\frac{{I}_{538}}{{I}_{548}}=B\,\exp \left(-\frac{\Delta E}{kT}\right),$$

where, I_538_ and I_548_ are the integrated intensities corresponding to the ^5^F_4_ → ^5^I_8_ and ^5^S_2_ → ^5^I_8_ transitions, respectively; B is dependent on radiative probabilities, degeneracies and emitted photon energies of the associated levels; ΔE is the energy gap between the ^5^F_4_ and ^5^S_2_ levels; k is the Boltzmann constant and T is the absolute temperature. Figure 4b shows the variation of FIR with temperature. The fitting of experimental data according to Eq. (13), gives B = 0.75 ± 0.01 and ΔE = 120 ± 10 cm^−1^.

Being an important parameter of sensor materials, the sensitivity (absolute) has been calculated as the rate of change of FIR with temperature and can be described as^51,52^

14 $$S=\frac{\partial (FIR)}{\partial T}=FIR\times \frac{\Delta E}{k{T}^{2}},$$

where all the terms have their usual meanings as defined in Eq. (13). The values of FIR and ΔE as obtained above were used to calculate the S-value. The sensor sensitivity, as presented in Fig. 4c as a function of temperature, confers that the sensitivity of the material is reasonably good and it decreases rapidly as temperature rises towards room temperature. A maximum value of S ~ 0.0095 K^−1^ is thus found at 12 K, which is decreased to 0.0002 K^−1^ at 300 K. Eventually, the material is very well sensitive to temperature especially at low temperatures. A similar trend of thermal S variation was observed for YVO_4_:Ho^3+^/Yb^3+^ as on excited by an ultraviolet light of 266 nm wavelength in our recent studies^28^.

### Site-selective and phase dependent time-resolved luminescence

**Figure 5:**
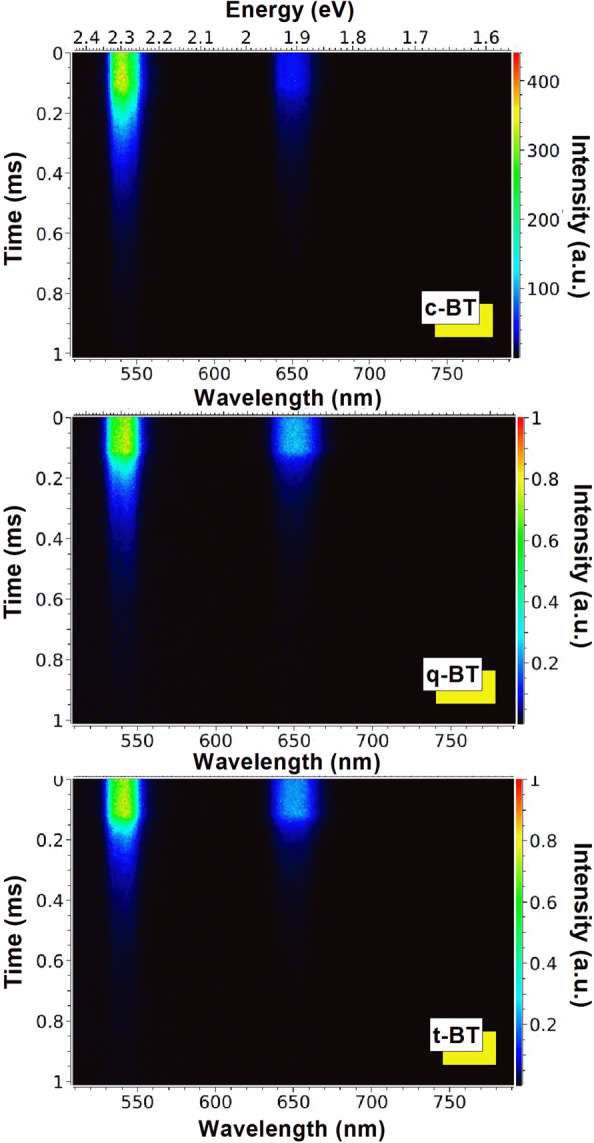
Streak camera evolved images of Ho^3+^-Yb^3+^: BaTiO_3_ in c-BT, q-BT and t-BT phases.

**Figure 6:**
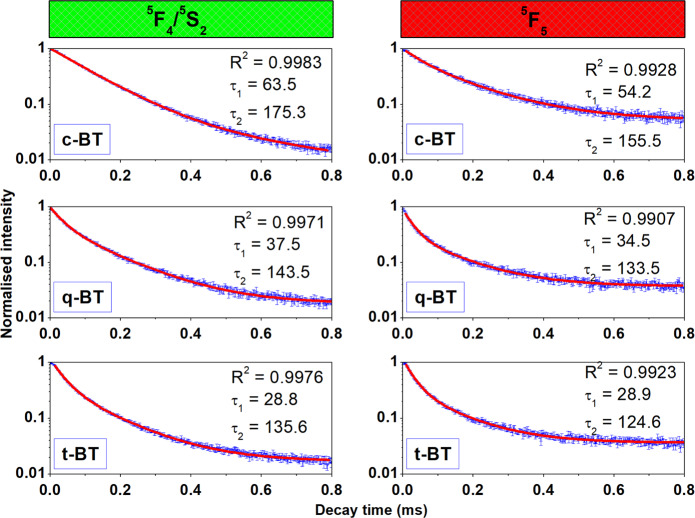
Time-resolved luminescence decay transients of ^5^F_4_/^5^S_2_ and ^5^F_5_ levels of Ho^3+^ in Ho^3+^-Yb^3+^: BaTiO_3_ upon a 980 nm laser-light excitation at room temperature. τ_1,2_ values in the insets are in μs.

In 1965, a preliminary report^53^ on emission spectra of Sm^3+^ in BaTiO_3_ had described two separate series of spectral bands in the Sm^3+^ present in two nonequivalent sites in the lattice of Ba^2+^ and Ti^4+^ sites. Therefore, in order to examine the variation of lifetime of the green upconversion (^5^F_4_/^5^S_2_) and red upconversion (^5^F_5_) emitting levels and to light on the site selective occupancy of Ho^3+^ ions in a BaTiO_3_ lattice, the time-resolved spectroscopic measurements were conducted by exciting the selective specimens with a 980 nm laser-light. The streak camera images (survey spectra) related to the ^5^F_4_/^5^S_2_ → ^5^I_8_ and ^5^F_5_ → ^5^I_8_ transitions of Ho^3+^ ions are portrayed in Fig. 5. The decay profiles of ^5^F_4_/^5^S_2_ and ^5^F_5_ levels (Fig. 6) can be described in terms of an empirical relation as follows^54^^,^

15 $$I(t)={I}_{0}+{A}_{1}{e}^{-t/{\tau }_{1}}+{A}_{2}{e}^{-t/{\tau }_{2}},$$

where I(t) and I_0_ are the upconversion intensities at time ‘t’ and ‘0’ (zero), respectively, with A_1_ and A_2_ as the fitting parameters at fast τ_1_ and slow τ_2_ components of the luminescence lifetimes. The values of decay times so calculated are summarized in Table 1 for c-BT, q-BT and t-BT samples.

**Table 1:** Short and long components of lifetime of ^5^F_4_/^5^S_2_ and ^5^F_5_ levels of Ho^3+^ ions co-doped in BaTiO_3_ of different phases.

Energy state	Lifetime components	Ho^3+^-Yb^3+^:BaTiO_3_		
		c-BT (μs)	q-BT (μs)	t-BT (μs)
^5^F_4_/^5^S_2_	τ_1_	63.5	37.5	28.8
	τ_2_	175.3	143.5	135.6
^5^F_5_	τ_1_	54.2	34.5	28.9
	τ_2_	155.5	133.5	124.6

Reported values have an error bar ≤ 2%.

The fraction (*ϕ*) of site-occupancy of Ho^3+^ ions of a characteristic lifetime value can be determined in a formula^16,55^,

16 $$\phi =\frac{{A}_{i}{\tau }_{i}}{\sum _{i=1,2}{A}_{i}{\tau }_{i}}\times 100.$$

The short-lived and long-lived components of lifetime can be interpreted by considering the existence of two types of emitting sites in a Ho^3+^-Yb^3+^: BaTiO_3_ sample. The Ho^3+^ (ionic size: 0.901 Å) can occupy the Ba^2+^ and Ti^4+^ sites with two unequal co-ordinations at the two sites; which in turn adapt different modulated lifetimes. As we know, the co-ordination numbers of Ba^2+^ and Ti^4+^ ions thereby are 12 and 6, respectively (Figure S8), which render a large difference in ionic size of Ho^3+^ and 12-coordinated Ba^2+^ sites, so as Ho^3+^ occupied Ba^2+^ sites form largely distorted octahedrons.

Conversely, if a produced defect in a charge difference is large, the local site is expected to lose its inversion symmetry. Nonetheless, while a Ho^3+^ ion occupies a 6-coordinated Ti^4+^ site, the ionic size difference is smaller over the former one and results in a lesser lattice distortion. In general, a comparatively short component of lifetime is associated with a low symmetric position of largely relaxed electronic transition rules, while a long component of lifetime is usually attributed to a relatively high symmetric position owing to the forbidden 4f-4f transitions^55^. Therefore, in this purview, it is proposed that the short component of lifetime arises in Ho^3+^ occupied Ti^4+^ sites (6-coordinated), while the long component of lifetime arises in Ho^3+^ occupied Ba^2+^ sites (12-coordinated). The fraction of Ho^3+^ ions at Ba^2+^ sites is calculated as 73% leaving behind a residual 27% value that in the Ti^4+^ sites in a c-BT sample. Further, as the Ho^3+^ site-occupancy reorders in a phase transition, it becomes 64% and 36% in the respective sites in a t-BT sample. A due change in the coordination numbers with a crystal phase is responsible for switching the site-occupancy, which eventually tunes local crystal fields around the R^3+^ carriers. It is also possible that part of Ho^3+^ ions occupies Ti^4+^ interstitial sites and some others occupy the surfaces in the small crystallites. The site occupancy of Sm^3+^ ions is reported in a similar SrZrO_3_ host^55^ in which Sr^2+^ sites serve as a minor and Zr^4+^ sites as a major host of Sm^3+^ dopants. In another example, Eu^3+^ ions doped in α-Zn_2_P_2_O_7_^56^ yield short and long lifetimes as they order at two differently coordinated Zn-sites.

### Second harmonic generation

**Figure 7:**
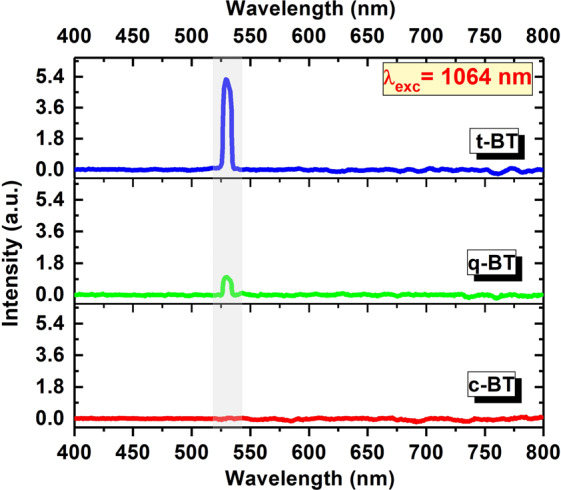
Second harmonic emission spectra of c-BT, q-BT and t-BT upon exciting with a 1064 nm pulsed laser (7 ns pulse width).

In SHG, two identical photons effectively combine giving rise to a single photon of twice the energy of the initial value. Nanocrystals exhibiting SHG are nowadays emerging in a versatile optical probe^57^. These nanocrystals are comprised of non-centrosymmetric crystallites and their overall contribution from the asymmetric unit cells give the SHG. Using SHG, BT nanocrystals have recently been demonstrated in stem cell labelling with high contrast images^58^. Thus, a phase transformation in Ho^3+^-Yb^3+^: BaTiO_3_ is not probed only in terms of its XRD but also its second harmonic signal. The magnitude of eccentricity of distortion in the crystal system is correlated with the intensity of the SHG signal. The SHG under a 1064 nm laser-light excitation for three BT specimens, which were annealed at 873 K (c-BT), 1173 K (q-BT) and 1473 K (t-BT), are shown in Fig. 7.

The presence of a green light at 532 nm in a half of the IR excitation wavelength supports the exploration of a non-linear effect in the BT samples. A markedly enhanced SHG signal intensity is exhibited for t-BT specimen. No such signal is visible in c-BT in view of its center of symmetry. As an eccentric distortion rises-up, the SHG turns-up in a t-BT phase. Our results of SHG, energy-upconversion of luminescence, and XRD patterns are consistent one another, and thus infer that our approach would lend itself for the use of a ‘structural probe’ employing the upconversion luminescence for identifying a crystal phase of the Ho^3+^-Yb^3+^ doped BTs.

## Conclusions

In conclusion, upconverted light emission from Ho^3+^-Yb^3+^: BaTiO_3_ is studied with the steady-state and time-resolved photoluminescence properties. The green to red emission intensity ratio varies across a phase transition of the crystallites on a due change in Ho^3+^ positions in the final lattice, with modified population of the Ho^3+^ energy levels. The experimental results on the number of photons involved in the upconversion processes were described in support with the theoretical approach of the rate equations. Effect of thermal annealing of the samples has been illustrated based on the Ho^3+^ emission spectra and the rate equations. It is found that the SHG signal intensity is a measure of eccentricity present in the non-centro-symmetric BaTiO_3_ crystals. Further, an analysis of decay time of ^5^F_4_/^5^S_2_ and ^5^F_5_ levels exhibits double decay characteristic of these levels. The Ho^3+^ occupancies of 73% and 27% found in the Ba^2+^ and Ti^4+^ sites in a cubic phase get modified to 64% and 36%, respectively, as it transforms to a tetragonal phase. The temperature dependent luminescence of this material can be used as a low-temperature probe, with a sensitivity as much as 0.0095 K^−1^ found at 12 K. Furthermore, consistent with XRD and SHG results, this work suggests a general approach to use upconversion luminescence of these samples in identifying their crystal structure and phase transitions.

## Methods and analyses

### Synthesis of Ho^3+^-Yb^3+^: BaTiO_3_ small crystallites

A doped BaTiO_3_ with Ho^3+^-Yb^3+^ ions, was prepared via a wet-chemical co-precipitation method of an optimized composition, 96.8 mol% BaTiO_3_ + 0.2 mol% Ho_2_O_3_ + 3.0 mol% Yb_2_O_3_, suitable for the upconversion emission^28,46^.

In a typical reaction batch^16,59^, BaCO_3,_ acetic acid (CH_3_COOH), titanium tetra-isopropoxide C_12_H_28_O_4_Ti, ytterbium acetate Yb(CH_3_COO)_3_ and holmium acetate Ho(CH_3_COO)_3_ of purity better than 99% were used as raw materials. A 1.5 g of barium carbonate was dissolved in 15 mℓ of acetic acid and warmed on a hot-plate of a magnetic stirrer at 80 °C, and then, cooled down to room temperature. Required amounts of 0.2 mol% of holmium acetate and 3.0 mol% of ytterbium acetate were added to this precursor in a homogeneous solution. As a source of Ti^4+^ ions, 2.0 mℓ titanium tetra-isopropoxide was mixed in the above solution and stirred on a magnetic stirrer for 1 h. At the final stage, 10 mℓ of distilled water was added to this solution for precipitating small slurries capping the cations of hydroxides, which were recovered and finally washed repeatedly in ethanol. So obtained sample was dried in ambient atmosphere over days and then annealed at 873, 1173 and 1473 K for 6 h, here after called as c-BT, q-BT and t-BT, respectively.

### Light-emission and other measurements

The XRD patterns were measured on a Bruker D8 Advance X-ray diffractometer using Cu-K_α_ (1.5405 Å) radiation. The absorption spectrum was taken in a diffuse reflectance mode using a Lambda 950, UV-Vis-NIR spectrophotometer (Perkin Elmer). The upconversion emission spectra at different pulse energies were recorded using a 980 nm diode laser on SP2300 grating spectrometer (Princeton Instruments, USA)^51^. The measurements of temperature-dependent upconversion spectra from 12 K to 300 K were performed on a SPEX 1000 M spectrometer^28^. A He-closed-cycle refrigerator at 10^−7^ mbar pressure was used to cool down the sample in a chamber. The time-resolved spectroscopic measurements were carried out under excitation at a 980 nm laser-light by a Ti-sapphire laser, Mira 900-F (Coherent) pumped by Verdi 10–532 nm laser with an experimental set up consisting of a streak camera (Hamamatsu C10910), a synchronous delay generator (Hamamatsu C10647-01), water-cooled CCD (Hamamatsu Orca R2), and a delay unit (Hamamatsu C1097-05), as used in our earlier studies^16,28^. Furthermore, a chopper wheel at 200 Hz frequency was employed for generating laser ‘pulses’. The SHG measurements were performed using a 1064 nm Nd-YAG laser of a pulse width of 7 ns (laser model: SplitLight 600, InnoLas Laser GmbH, Germany). A suitable filter was used to block 1064 nm radiation to pass at the CCD detector.

## Supplementary information

Supplementary Information.

## Data Availability

The experimental data is available upon request from the corresponding author.

## References

[1] Ohtomo A, Muller DA, Grazul JL, Hwang HY (2002). Artificial charge-modulationin atomic-scale perovskite titanate superlattices. Nature.

[2] Tang P, Towner DJ, Meier AL, Wessels BW (2004). Low-voltage, polarization-insensitive, electro-optic modulator based on a polydomain barium titanate thin film. Appl. Phys. Lett..

[3] Scott JF (2007). Applications of modern ferroelectrics. Science.

[4] Xu, Y. Ferroelectric materials and their applications. Elsevier (2013).

[5] Ramadass N (1978). ABO_3_-type oxides—Their structure and properties—A bird’s eye view. Mater. Sci. Eng..

[6] Mueller V, Beige H, Abicht HP, Eisenschmidt C (2004). X-ray diffraction study revealing phase coexistence in barium titanate stannate. J. Mater. Res..

[7] Singh A, Patel JP, Pandey D (2009). High temperature ferroic phase transitions and evidence of paraelectric cubic phase in the multiferroic 0.8 BiFeO_3_–0.2 BaTiO_3_. Appl. Phys. Lett..

[8] Bhattacharjee S, Senyshyn A, Krishna PSR, Fuess H, Pandey D (2010). Simultaneous changes of nuclear and magnetic structures across the morphotropic phase boundary in (1−x)BiFeO_3_− xPbTiO_3_. Appl. Phys. Lett..

[9] Verma N, Singh S, Yadav BC (2014). Experimental investigations on barium titanate nanocomposite thin films as an opto-electronic humidity sensor. J. Exp. Nanosci..

[10] Sikarwar S, Yadav BC (2015). Opto-electronic humidity sensor: A review. Sens. Actuator A-Phys..

[11] Singh M, Yadav BC, Ranjan A, Sonker RK, Kaur M (2017). Detection of liquefied petroleum gas below lowest explosion limit (LEL) using nanostructured hexagonal strontium ferrite thin film. Sens. Actuator B-Chem..

[12] Manikandan V, Singh M, Yadav BC, Vigneselvan S (2018). Room-temperature gas sensing properties of nanocrystalline-structured indium-substituted copper ferrite thin film. J. Electron. Mater..

[13] Ye, Z. G. (Ed.). Handbook of advanced dielectric, piezoelectric and ferroelectric materials: Synthesis, properties and applications. Elsevier (2008).

[14] Yu K, Wang H, Zhou Y, Bai Y, Niu Y (2013). Enhanced dielectric properties of BaTiO_3_/poly (vinylidene fluoride) nanocomposites for energy storage applications. J. Appl. Phys..

[15] Xie L, Huang X, Huang Y, Yang K, Jiang P (2013). Core@ double-shell structured BaTiO3–polymer nanocomposites with high dielectric constant and low dielectric loss for energy storage application. J. Phys. Chem. C..

[16] Mahata MK (2015). Incorporation of Zn^2+^ ions into BaTiO_3_: Er^3+^/Yb^3+^ nanophosphor: an effective way to enhance upconversion, defect luminescence and temperature sensing. Phys. Chem. Chem. Phys..

[17] Mitoseriu L (1996). Grain size dependence of switching properties of ferroelectric BaTiO3 ceramics. Jpn. J. Appl. Phys..

[18] Ram S, Jana A, Kundu TK (2007). Ferroelectric BaTiO_3_ phase of orthorhombic crystal structure contained in nanoparticles. J. Appl. Phys..

[19] Jana A, Ram S, Kundu TK (2007). BaTiO_3_ nanoparticles of orthorhombic structure following a polymer precursor. Part I. X-ray diffraction and electron paramagnetic resonance. Philos. Mag..

[20] Tan Y (2015). Unfolding grain size effects in barium titanate ferroelectric ceramics. Sci. Rep..

[21] Yoneda Y, Sakaue K, Terauchi H (2001). Phase transition of BaTiO_3_ thin films. J. Phys. Condens. Mat..

[22] Tsur Y, Dunbar TD, Randall CA (2001). Crystal and defect chemistry of rare earth cations in BaTiO_3_. J. Electroceram..

[23] Tsur Y, Hitomi A, Scrymgeour I, Randall CA (2001). Site occupancy of rare-earth cations in BaTiO_3_. Jpn. J. Appl. Phys..

[24] Auzel F (2004). Upconversion and anti-stokes processes with f and d ions in solids. Chem. Rev..

[25] Ram, S., Mishra, A., & Fecht, H. J. Radiative emissions in rare-earth ions in Al_2_O_3_ and nanocomposites. In Encyclopedia of Nanoscience and Nanotechnology (Vol. 22, No. 288, pp. 179-288). American Scientific Publishers (2011).

[26] Mahata, M. K., Hofsäss, H. C. & Vetter, U. Photon-upconverting materials: advances and prospects for various emerging applications (pp. 109–131). Luminescence: An Outlook on the Phenomena and their Applications (ed. J. Thirumalai). InTech (2016).

[27] Wen S (2018). Advances in highly doped upconversion nanoparticles. Nat. Commun..

[28] Mahata MK, Koppe T, Kumar K, Hofsäss H, Vetter U (2016). Demonstration of temperature dependent energy migration in dual-mode YVO_4_: Ho^3+^/Yb^3+^ nanocrystals for low temperature thermometry. Sci. Rep..

[29] Gorris HH, Resch-Genger U (2017). Perspectives and challenges of photon-upconversion nanoparticles-Part II: bioanalytical applications. Anal. Bioanal. Chem..

[30] Soni AK, Mahata MK (2017). Photoluminescence and cathodoluminescence studies of Er^3+^-activated strontium molybdate for solid-state lighting and display applications. Mater. Res. Express.

[31] Tiwari SP (2018). Future prospects of fluoride based upconversion nanoparticles for emerging applications in biomedical and energy harvesting. J. Vac. Sci. Technol. B.

[32] Kumar V, Pandey A, Ntwaeaborwa OM, Swart HC (2018). Energy transfer upconversion in Er^3+^-Tm^3+^ codoped sodium silicate glass. Physica B..

[33] Balakrishna A (2018). Synthesis, structure and optical studies of ZnO: Eu^3+^, Er^3+^, Yb^3+^ thin films: Enhanced up-conversion emission. Colloid. Surf. A.

[34] Chen G, Qiu H, Prasad PN, Chen X (2014). Upconversion nanoparticles: design, nanochemistry, and applications in theranostics. Chem. Rev..

[35] Zhou B, Shi B, Jin D, Liu X (2015). Controlling upconversion nanocrystals for emerging applications. Nat. Nanotechnol..

[36] Maczka M (2010). Lattice dynamics and pressure-induced phase transitions in Bi_2_W_2_O_9_: High-pressure Raman study. Phys. Rev. B.

[37] Yao Y, Luo L, Li W, Zhou J, Wang F (2015). An intuitive method to probe phase structure by upconversion photoluminescence of Er^3+^ doped in ferroelectric Pb (Mg_1/3_Nb_2/3_)O_3_-PbTiO_3_. Appl. Phys. Lett..

[38] Sukul PP, Mahata MK, Ghorai UK, Kumar K (2019). Crystal phase induced upconversion enhancement in Er^3+^/Yb^3+^ doped SrTiO3 ceramic and its temperature sensing studies. Spectrochim. Acta A.

[39] Ohara Y, Koumoto K, Yanagida H (1985). Barium titanate ceramics with high piezoelectricity fabricated from fibrous particles. J. Am. Ceram. Soc..

[40] Li B, Zhang S, Zhou X, Chen Z, Wang S (2007). Microstructure and dielectric properties of Y/Zn codoped BaTiO_3_ ceramics. J. Mater. Sci..

[41] Sengodan R, Shekar B, Chandar Sathish S (2014). Structure, surface morphology and optical properties of BaTiO_3_ powders prepared by wet chemical method. Indian J. Pure Appl. Phys..

[42] Scherrer P (1918). Bestimmung der Größe und der inneren Struktur von Kolloidteilchen mittels Röntgenstrahlen. Nachrichten von der Gesellschaft der Wissenschaften zu Göttingen, Mathematisch-Physikalische Klasse.

[43] Chen YF, Lee CY, Yeng MY, Chiu HT (2003). The effect of calcination temperature on the crystallinity of TiO_2_ nanopowders. J. Cryst. Growth.

[44] Rao F, Kim M, Freeman AJ, Tang S, Anthony M (1997). Structural and electronic properties of transition-metal/BaTiO_3_(001) interfaces. Phys. Rev. B.

[45] Pollnau M, Gamelin DR, Luethi SR, Guedel HU (2000). Power dependence of upconversion luminescence in lanthanide and transition-metal-ion systems. Phys. Rev. B.

[46] Mahata MK, Koppe T, Hofsäss H, Kumar K, Vetter U (2015). Host sensitized luminescence and time-resolved spectroscopy of YVO4: Ho^3+^ nanocrystals. Phys. Procedia.

[47] Zhang F, Li G, Zhang W, Yan YL (2015). Phase-Dependent Enhancement of the Green-Emitting Upconversion Fluorescence in LaVO4: Yb^3+^, Er^3+^. Inorg. Chem..

[48] Zuo Q, Luo L, Li W, Wang F (2016). An effective method to detect the Curie transition of Er^3+^/Yb^3+^ co-doped BaTiO_3_ ceramics by up-conversion photoluminescence intensity ratio. J..

[49] Mahata MK, Kumari A, Rai VK, Kumar K (2013). Er^3+^, Yb^3+^ doped yttrium oxide phosphor as a temperature sensor. AIP Conf. Proc..

[50] Sinha S, Mahata MK, Kumar K (2016). Up/down-converted green luminescence of Er^3+^–Yb^3+^ doped paramagnetic gadolinium molybdate: a highly sensitive thermographic phosphor for multifunctional applications. RSC Adv..

[51] Mahata MK, Kumar K, Rai VK (2015). Er^3+^-Yb^3+^ doped vanadate nanocrystals: a highly sensitive thermographic phosphor and its optical nanoheater behavior. Sensor. Actuat. B-Chem..

[52] Sinha S, Mahata MK, Kumar K (2018). Comparative thermometric properties of bi-functional Er^3+^–Yb^3+^ doped rare earth (RE = Y, Gd and La) molybdates. Mater. Res. Express.

[53] Makishim S, Yamamoto H, Tomotsu T, Shionoya S (1965). Luminescence spectra of Sm^3+^ in BaTiO_3_ host lattice. J. Phys. Soc. Jpn..

[54] Sinha S, Mahata MK, Kumar K (2019). Enhancing the upconversion luminescence properties of Er^3+^–Yb^3+^ doped yttrium molybdate through Mg^2+^ incorporation: effect of laser excitation power on temperature sensing and heat generation. New J. Chem..

[55] Gupta SK, Ghosh PS, Pathak N, Aryab A, Natarajana V (2014). Understanding the local environment of Sm^3+^ in doped SrZrO_3_ and energy transfer mechanism using time-resolved luminescence: a combined theoretical and experimental approach. RSC Adv..

[56] Gupta SK, Mohapatra M, Godbole SV, Natarajan V (2013). On the unusual photoluminescence of Eu^3+^ in α-Zn_2_P_2_O_7_: a time resolved emission spectrometric and Judd-Ofelt study. RSC Adv..

[57] Kim E (2013). Second-harmonic generation of single BaTiO_3_ nanoparticles down to 22 nm diameter. ACS Nano.

[58] Sugiyama N, Sonay AY, Tussiwand R, Cohen BE, Pantazis P (2018). Stem Cell Imaging: Effective Labeling of Primary Somatic Stem Cells with BaTiO_3_ Nanocrystals for Second Harmonic Generation Imaging. Small.

[59] Mahata MK, Kumar K, Rai VK (2014). Structural and optical properties of Er^3+^/Yb^3+^ doped barium titanate phosphor prepared by co-precipitation method. Spectrochim. Acta A.

