# Supplementary material for: Upconversion photoluminescence of Ho3+-Yb3+ doped barium titanate nanocrystallites: Optical tools for structural phase detection and temperature probing
Source: Sci Rep. 2020 May 29;10:8775. doi: 10.1038/s41598-020-65149-z (PMC7260367; doi:10.1038/s41598-020-65149-z)
Supplement: Supplementary file 1 — Supplementary Information. [file 41598_2020_65149_MOESM1_ESM.docx]

**Supplementary Information**

**Upconversion photoluminescence of Ho^3+^-Yb^3+^ doped barium titanate nanocrystals: Optical tools for structural phase detection and temperature probing**

**Manoj Kumar Mahata^1, *^, Tristan Koppe^1^, Kaushal Kumar^2^, Hans Hofsäss^1^ & Ulrich Vetter^1^**

*^1^Second Institute of Physics, University of Goettingen, Friedrich-Hund-Platz 1, 37077 Göttingen, Germany*

*^2^Department of Physics, Indian Institute of Technology (Indian School of Mines), Dhanbad-826004, India*

[**mmahata@uni-goettingen.de*](mailto:*mmahata@uni-goettingen.de)

**Optical properties**:

The near-infrared emission band in upconversion spectrum (Fig. 2) between 730-780 nm is consisted of two emission peaks which are centered at 747 and 756 nm. These bands are attributed to ^5^F_4_, ^5^S_2_→^5^I_7_ transition (Figure 2b). The red emission band between 625-690 nm is attributed to the ^5^F_5_→ ^5^I_8_ transition. The band showing highest emission intensity is composed of two emission peaks at 538 and 548 nm, corresponding to ^5^F_4_, ^5^S_2_→^5^I_8_ transitions of Ho^3+^ ions. It is worthy to note that the intensity of green band is 4 and 97.5 times higher than that of the red and NIR emission bands, respectively.

Apart from these, appearance of a series of low intense emission bands at 394, 424 and 464 nm can be attributed to the ^5^G_4_, ^3^K_7_→ ^5^I_8_; ^5^G_5_→ ^5^I_8_ and ^5^F_1_, ^5^G_6_→ ^5^I_8_ transitions, respectively of Ho^3+^ (Figure S4).

Between the two basic population mechanisms, involved in the upconversion processes, namely, energy transfer upconversion (ET) and excited state absorption (ESA), the ET process plays a substantial role in upconversion mechanism owing to the larger absorption cross-section of Yb^3+^ ions at the excitation wavelength- 980 nm. The green upconversion is involved with two consecutive ETs from Yb^3+^ to Ho^3+^ ion. The Yb^3+^ ions are initially excited from ^2^F_7/2_ to ^2^F_5/2_ level via absorbing a 980 nm photon and then transfer its energy to the ^5^I_6_ level of a nearby Ho^3+^ ion by ET-1 process. It is noteworthy that owing to the slightly lower energy of ^5^I_6_ of Ho^3+^ than the ^2^F_5/2_ level of Yb^3+^, phonons are believed to partake in the energy transfer process. Subsequently, ET-2 occurs from ^5^I_6_ level by exciting Ho^3+^ ion to ^5^F_4_, ^5^S_2_ levels while the excess energy ~600 cm^-1^ is taken up as phonon energy by the barium titanate lattice. Meantime, a major part of the Ho^3+^ ions at ^5^F_4_ /^5^S_2_ decays radiatively to the ^5^I_8_ level, resulting the green emission (538 nm - 548 nm) whereas rest of the part depopulates at ^5^I_7_ level, producing the NIR emission (755 nm). Appearance of a greenish blue emission band peaking at 489 nm can be attributed to the transition of Yb^3+^ or Ho^3+^ ion [1-3]

For the red upconversion emission, population of ^5^F_5_ level takes place through two possible ways: ET-3 from ^5^I_7_ level and non-radiative decay from ^5^S_2_ level just after ET-2 process. Moreover, estimation of the energy gap from emission spectrum, between the ^5^S_2_ and ^5^F_5_ levels as ~3021 cm^-1^, points out the requirement of 4-5 phonons to bridge up the energy gap, indicating a probable way of non-radiative transition from ^5^S_2_ to ^5^F_5_ level. To populate the ^5^F_4_ /^5^S_2_ level, at least two excitation photons are required while for the levels above the ^5^F_4_ /^5^S_2_, more than two photons are needed (Figure 2b). The Ho^3+^ ions at the ^5^F_4_ /^5^S_2_ level absorb photon through another ET process from Yb^3+^ ion to reach at the ^5^G_3_ level, populating the levels lying between ^5^G_3_ and ^5^G_6_ through phonon relaxation and produces the emission bands located at 394, 424 and 464 nm (Figure S4).

**Supplementary Figures**


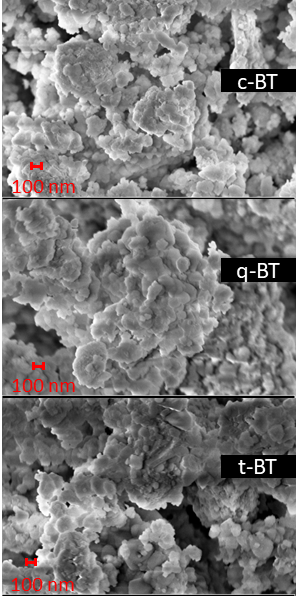


Fig. S1. Field emission scanning electron microscope images of Ho^3+^/Yb^3+^ doped BaTiO_3_: c-BT (upper), q-BT (middle), and t-BT (lower).


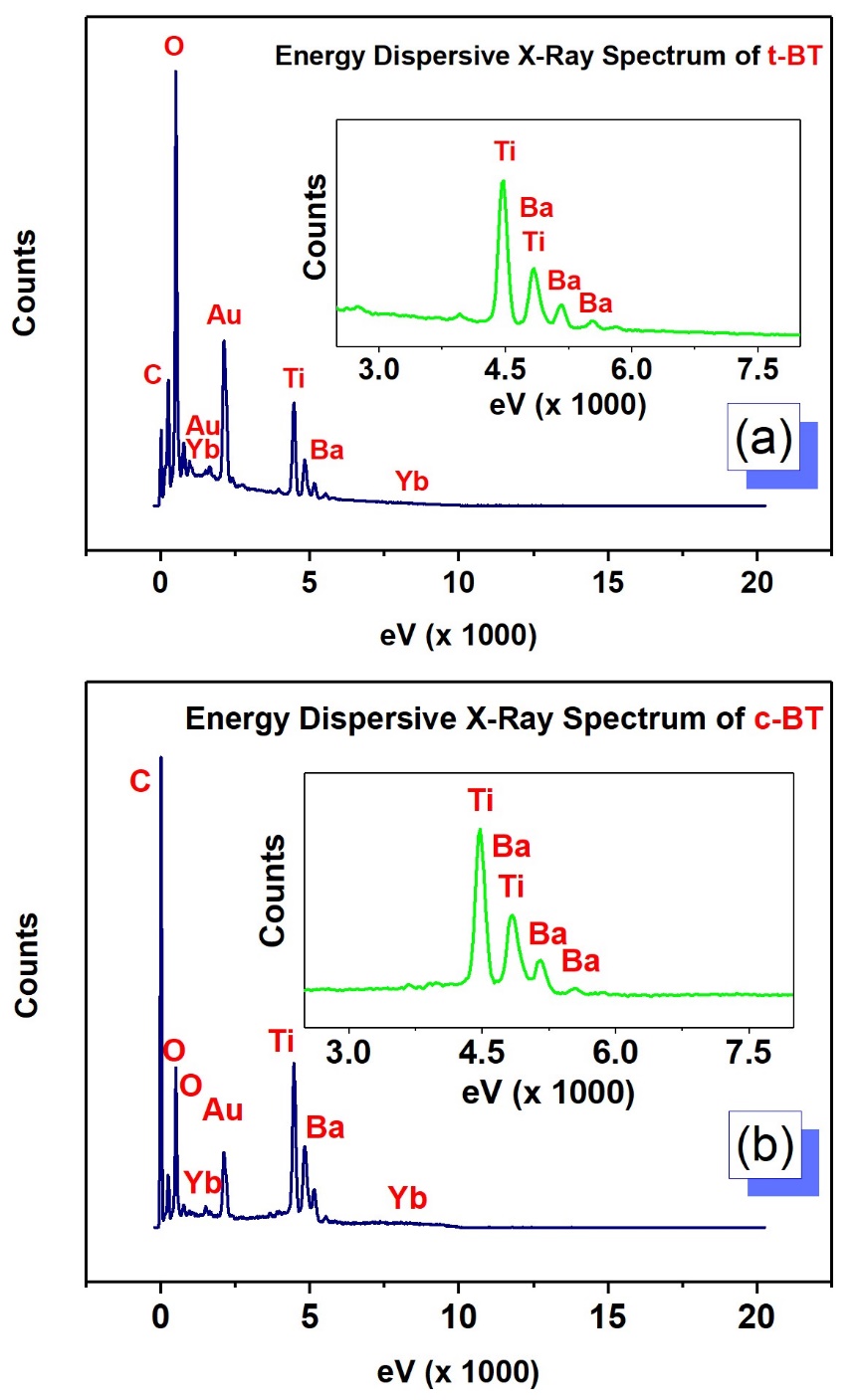


Fig. S2. Energy-dispersive X-ray spectrum of (a) t-BT, and (b) c-BT. Insets show the enlarged part within 2500-8000 eV.


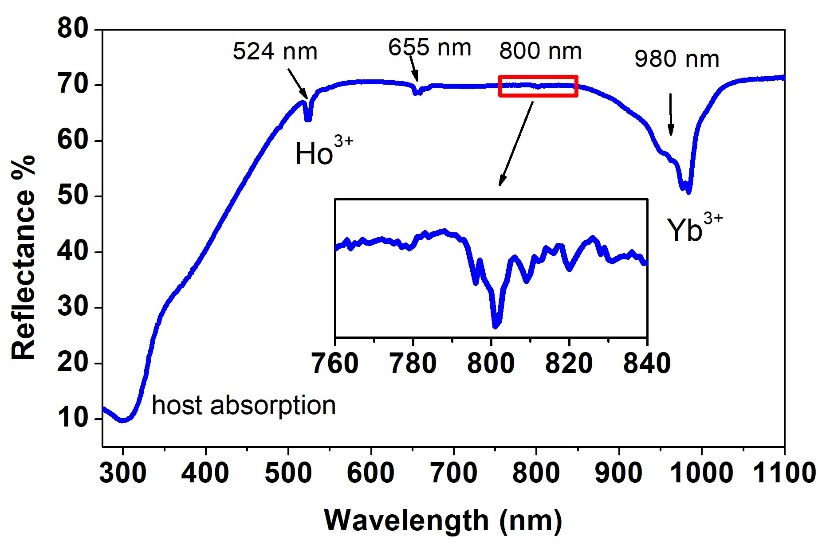


Fig. S3. Absorption spectrum in diffuse reflectance mode of Ho^3+^-Yb^3+^ doped BaTiO_3_ particles (t-BT). The band around 300 nm is due to the optical band gap of the material.


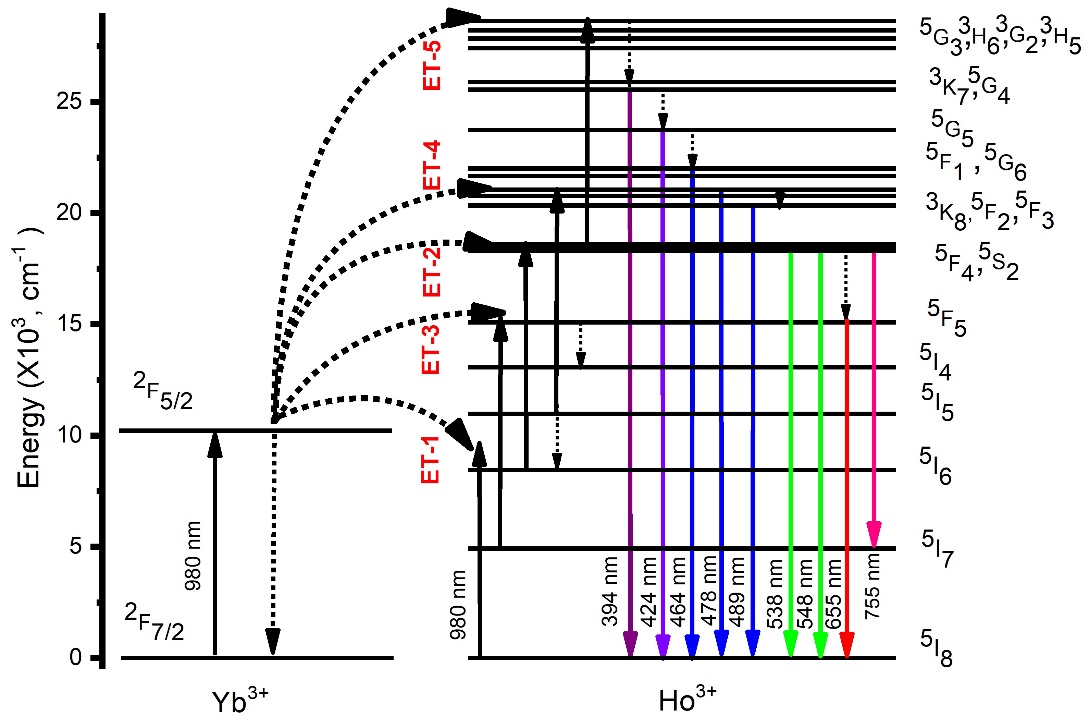


Fig. S4. The energy level diagram with possible transition pathways in Ho^3+^-Yb^3+^ system in BaTiO_3_.


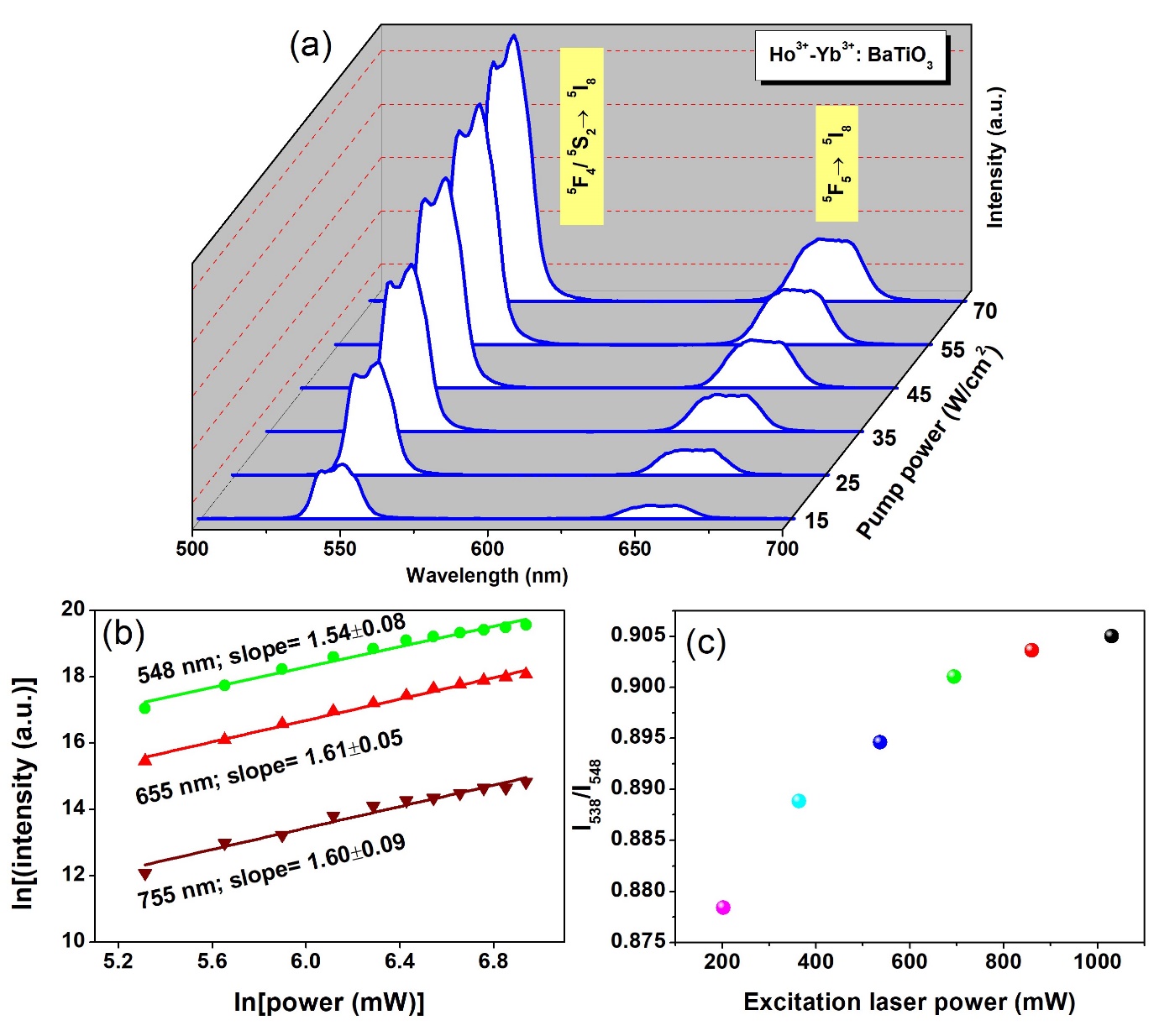


Fig. S5(a). Variation of upconversion emission intensity with different excitation power densities, (b) ln(I)-ln(P) plots of 548, 655 and 755 nm emission bands, giving the number of photons participated in the upconversion processes, and (c) variation of ratio of 538 nm (^5^F_4_→ ^5^I_8_) to 548 nm (^5^S_2_ → ^5^I_8_) emission band with laser excitation power in t-BT (Ho^3+^-Yb^3+^: BaTiO_3_ annealed at 1473 K)

**
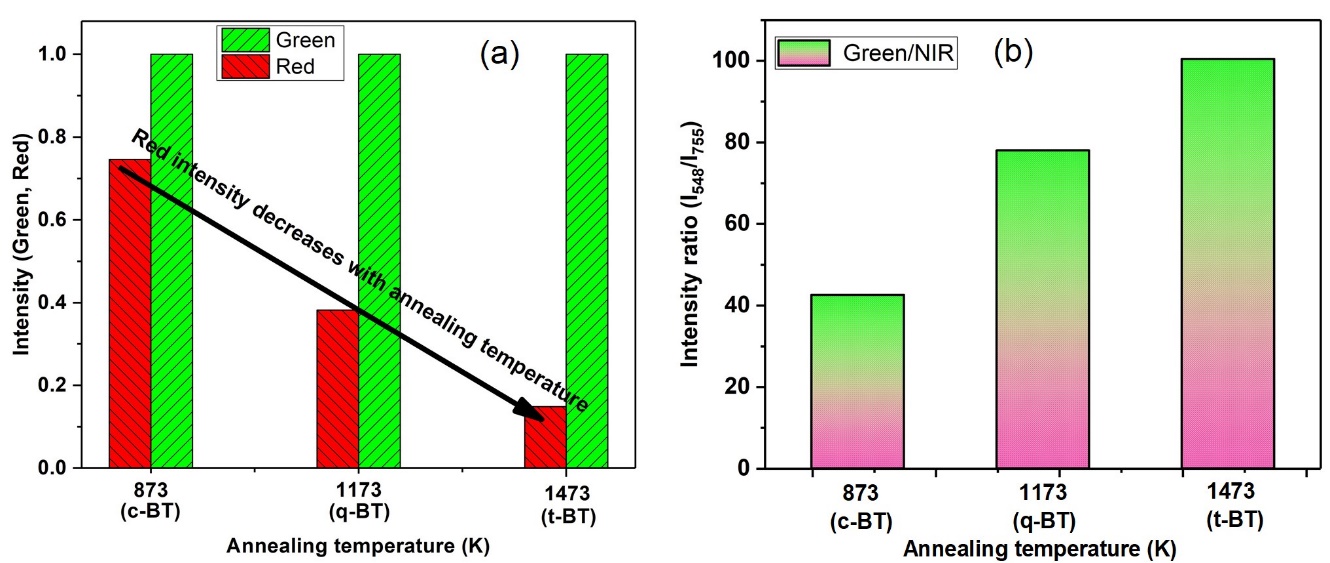
**

Fig. S6(a). Variation of green (^5^F_4_/^5^S_2_→ ^5^I_8_) and red (^5^F_5_→ ^5^I_8_) emission intensity of three samples with annealing temperature (green intensity is normalized), and (b) ratio of 548 nm (^5^F_4_/^5^S_2_→ ^5^I_8_) to 755 nm (^5^F_4_/^5^S_2_→ ^5^I_7_) band at three structural phases of barium titanate.

**
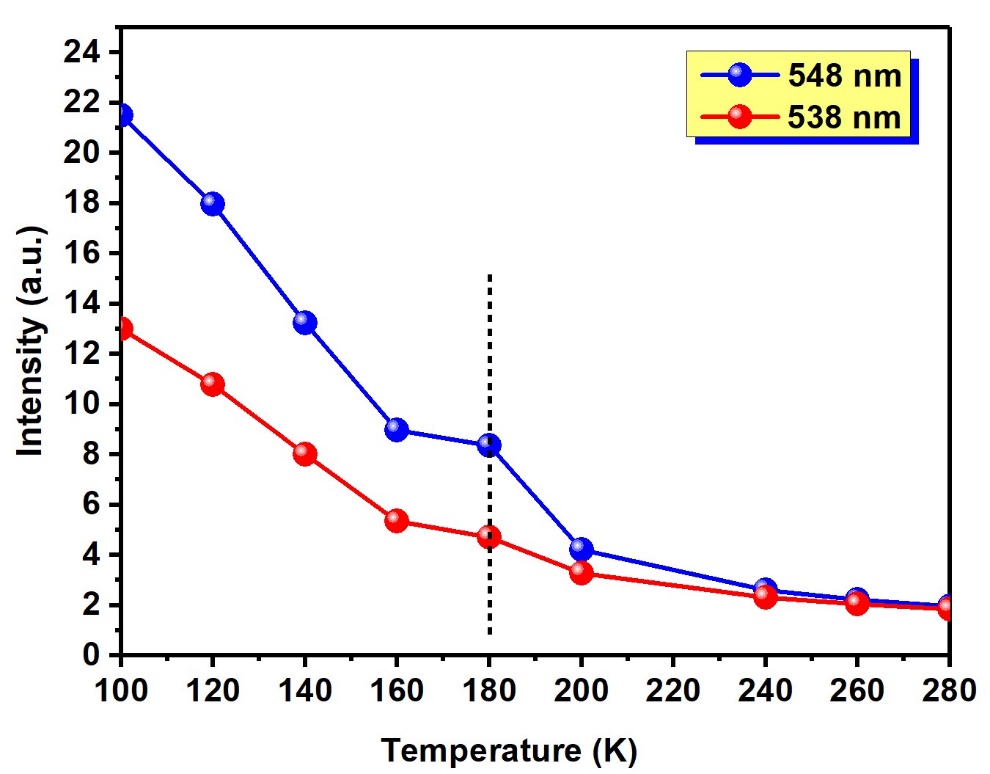
**

**Fig. S7:** Variation of 538 nm (^5^F_4_→ ^5^I_8_) and 548 nm (^5^S_2_ → ^5^I_8_) emission bands with temperature. At ∼180 K, the jump in the emission intensity is observed due to ferroelectric phase transition of barium titanate. As the temperature increases, the emission intensities of both the bands tend to be equal.


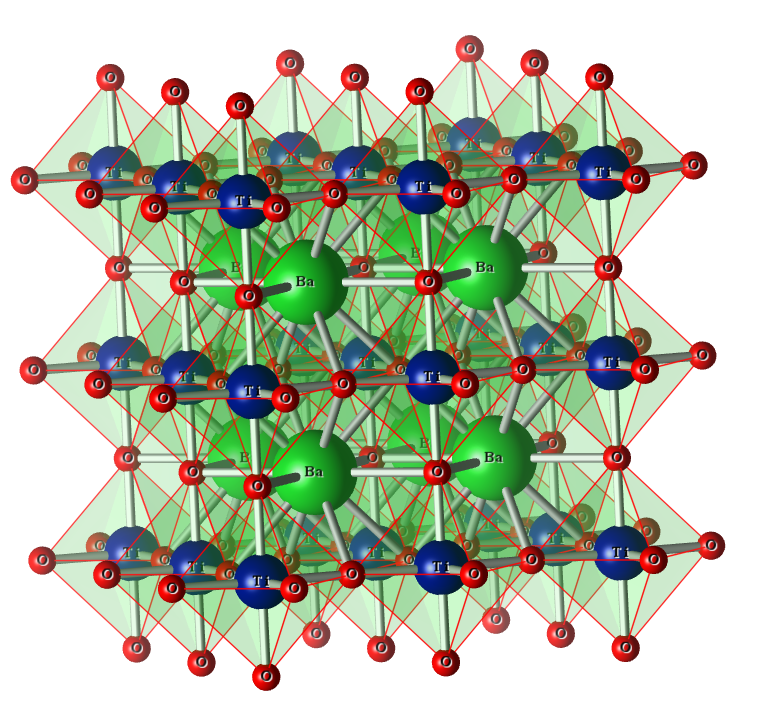


**Fig. S8:** A 2 × 2 × 2 cubic BaTiO_3_ super cell, with all octahedra. coordination of each cation has been shown by drawing the polyhedra. The Visualization for Electronic and Structural Analysis (VESTA) software [4] was used for drawing the structure.

**References**

1. Boulon, G. Why so deep research on Yb^3+^-doped optical inorganic materials. Journal of Alloys and Compounds, **451**, 1 (2008).
2. Teng, Y., Zhou, J., Liu, X., Ye, S., & Qiu, J. Efficient broadband near-infrared quantum cutting for solar cells. Optics Express, **18**, 9671 (2010).
3. Li, W., Cheng, J., Zhao, G., Chen, W., Hu, L., Guzik, M., & Boulon, G. Violet-green excitation for NIR luminescence of Yb^3+^ ions in Bi_2_O_3_-B_2_O_3_-SiO_2_-Ga_2_O_3_ glasses. Optics Express, **22**, 8831 (2014).
4. Momma, K., & Izumi, F. VESTA 3 for three-dimensional visualization of crystal, volumetric and morphology data. Journal of Applied Crystallography, 44(6), 1272-1276 (2011).
